# A New *In Vitro* Strand Transfer Assay for Monitoring Bacterial Class 1 Integron Recombinase IntI1 Activity

**DOI:** 10.1371/journal.pone.0001315

**Published:** 2007-12-19

**Authors:** Véronique Dubois, Carole Debreyer, Simon Litvak, Claudine Quentin, Vincent Parissi

**Affiliations:** Laboratory of Cellular and Molecular Microbiology and Pathogenicity (MCMP), UMR 5097-CNRS, University Victor Segalen Bordeaux 2, Bordeaux, France; The Scripps Research Institute, United States of America

## Abstract

IntI1 integrase is a tyrosine recombinase involved in the mobility of antibiotic resistance gene cassettes within bacterial class 1 integrons. Recent data have shown that its recombination specifically involves the bottom strand of the *attC* site, but the exact mechanism of the reaction is still unclear. An efficient *in vitro* assay is still required to better characterize the biochemical properties of the enzyme. In this report we describe for the first time an *in vitro* system partially reproducing the activity of a recombinant pure IntI1. This new assay, which constitutes the only available *in vitro* model of recombination by IntI1, was used to determine whether this enzyme might be the sole bacterial protein required for the recombination process. Results show that IntI1 possesses all the features needed for performing recombination between *attI* and *attC* sites. However, differences in the *in vitro* intermolecular recombination efficiencies were found according to the target sites and were correlated with DNA affinities of the enzyme but not with *in vivo* data. The differential affinity of the enzyme for each site, its capacity to bind to a single-stranded structure at the *attC* site and the recombination observed with single-stranded substrates unambiguously confirm that it constitutes an important intermediary in the reaction. Our data strongly suggest that the enzyme possesses all the functions for generating and/or recognizing this structure even in the absence of other cellular factors. Furthermore, the *in vitro* assay reported here constitutes a powerful tool for the analysis of the recombination steps catalyzed by IntI1, its structure-function studies and the search for specific inhibitors.

## Introduction

Since the introduction of antibiotics in the treatment of human infectious diseases, bacterial resistance has become an ever-increasing problem that threatens the clinical usefulness of these drugs. The prevalence of antibiotic resistance is mainly due to the horizontal transfer of antibiotic-resistance genes, conveyed by mobile genetic elements such as plasmids and transposons. Integrons are a class of site-specific recombination elements which insert and excise mobile antibiotic resistance gene cassettes, and which are located on plasmids and/or transposons. All class 1 integrons consist of two conserved sequences (CS) flanking a variable central region encompassing antibiotic resistance gene cassettes [1]. The highly conserved 5′CS includes an *intI* gene encoding an integrase, an adjacent recombination site *attI* and a promoter region, while the 3′CS is more variable. While several classes of integrons have been identified according to the type of integrase, the most prevalent class 1 integrons are characterized by an *intI1* gene encoding an integrase of 337 amino acids.

Gene cassettes can exist in two forms: either as free covalently closed supercoiled circular molecules that are unable to replicate, or as linear molecules integrated at the *attI* site into integrons [2]. Gene cassettes consist of a single coding sequence carrying at its 3′ end an *attC* recombination site. The *attC* sites, also called 59-base elements, are essentially formed from two imperfect inverted repeats with a 7 bp core site GTTRRRY in the right end consensus region which is essential for *in vivo* recombination [3]–[6].

The integrase is a member of the tyrosine recombinase family, which catalyzes cassette integration and excision by a site-specific recombination, occurring naturally between the *attI* of the integron and the *attC* of a gene cassette, or between two *attC* sites. Insertion can also take place, albeit rarely, at non-specific or secondary DNA sites which display sequence analogies with the core site [4], [7]. The *attI* site is 70 bp long and contains four IntI1 binding sites at −50, −30, −7 and 0 including the 7 base core region GTTRRRY[2], [4], [8]–[11]. The cross-over point occurs between the G base of a core site and the first T base of a second core site [7]–[12].

Until recently, the reaction catalyzed by the IntI1 integrase encoded by class 1 integrons has essentially been studied *in vivo.* In bacteria, IntI1 can catalyze recombination between either two *attC*, one *attI1* and one *attC*, or two *attI* sites [13]. Recent *in vivo* and structural data have provided important information on the mechanism by which recombination occurs in class 1 integrons. It has been clearly demonstrated that the *in vivo* recombination process involves the hairpin-folded bottom strand of *attC* [14]. The now available crystal structure of IntI from *Vibrio cholerae* bound to the bottom strand of *attC* site showed that DNA target site recognition and high-order synaptic assembly are not dependent on canonical DNA but on the position of two flipped-out bases that interact in *cis* and in *trans* with the integrase. These extrahelical bases originate from the folding of the bottom strand of *attC* due to its imperfect dyad symmetry [15]. All these new data confirm previous reports of the *in vitro* interaction between integrase and its DNA substrates [9], [16], [17]. Taken together these results support a new paradigm for how sequence-degenerate single-stranded genetic material is recognized and exchanged between bacteria.

Despite these functional and structural breakthroughs, several points remain obscure. Is the single-stranded intermediate *attC* generated during bacterial DNA replication or by IntI1 itself? Is IntI1 sufficient as sole bacterial protein for performing all the recombination steps or does it require other factors? These questions might be answered by carrying out an *in vitro* assay using recombinant pure enzyme. To date, however, all attempts to set up such experimental systems have been unsuccessful and no *in vitro* assay has been available, making it difficult to perform further biochemical analysis of the recombination mechanism.

**Figure 1:**
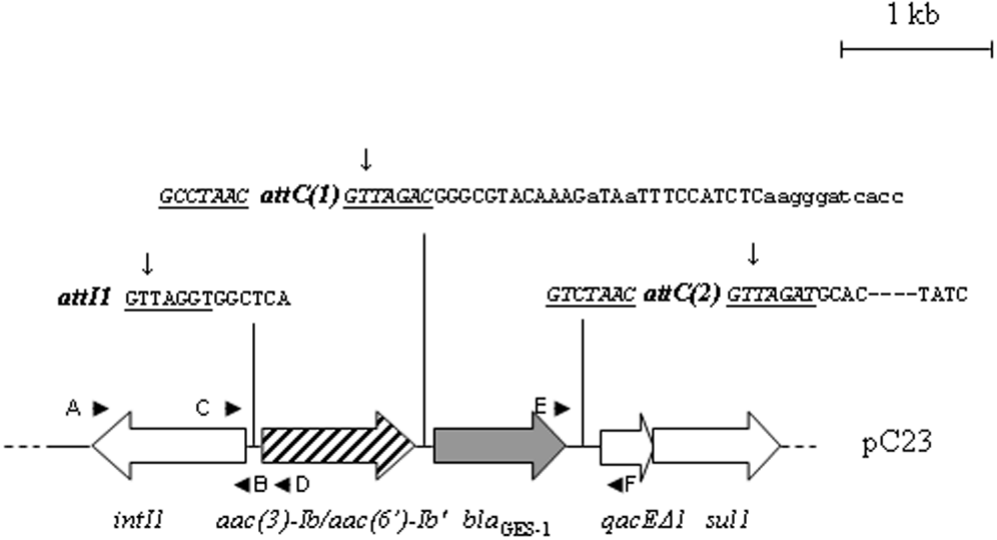
Schematic representation of recombinant plasmid pC23 part structure encoding the class 1 integron in *P. aeruginosa* Pa695 (adapted from Dubois et al., 2002, accession number AF355189). The horizontal arrows indicate the translation orientation of the genes. The conserved core and inverse core sites are underlined and the cassette boundaries are represented by vertical arrows. The black arrowheads indicate the different primers described in materials and methods used for cloning the IntI1 gene and the different recombination and excision substrates: A: IntI1-3′-stop, B: IntI1-5′-Topo, C: attI1-LBamH1, D: attI1-RHindIII, E: C12T3bis and F: 3′CS.

Thus, we sought to produce and purify an active recombinant integrase from a class 1 integron previously isolated from a clinical strain of *Pseudomonas aeruginosa* [18] see figure 1. Then we set up an *in vitro* recombination assay to characterize its biochemical properties. Using this new assay, we show that IntI1 possesses an *in vitro* recombination activity on both *attI1* and *attC* but with different efficiencies, consistent with its differential affinity for each DNA element. This new *in vitro* assay of IntI1 recombination activity allows further functional analysis of the protein.

## Results

### Expression, *in vivo* activity and purification of the recombinant IntI1 enzyme

The IntI1(his)_6_ recombinant protein was expressed from pET101D-Topo vector containing the complete gene encoding the *P. aeruginosa* IntI1 class 1 integron cloned as described in materials and methods. In the resulting pET101D-IntI1 vector, the IntI1 open reading frame was expressed from T7 promoter and fused to a poly(his)6 C-terminal tag. The activity of the fused enzyme was first checked by *in vivo* excision and recombination assays. In the presence of plasmid pSf2032 carrying an integrase-defective class 2 integron (whose *attI2* sequences were shown to be recognized by IntI1), the recombinant integrase was shown to be active for excision activity (40% of cassettes lost). Moreover, in the presence of pSf2032 and pACYC184 containing the specific *attI1* recombination sequence, recombination was observed at a rate of 4.4×10^5^. No excision or recombination events were detected in the absence of pET101D-IntI1 vectors. These results were consistent with previously described recombination rates [19] and demonstrated that the integrase IntI1 fused to the (his)6 tag was functional *in vivo* for all the activities expected of bacterial recombinase in cells. Importantly, the (his)_6_ tag did not interfere significantly with the catalysis, easily allowing us to purify an active enzyme and further characterize its *in vitro* properties.

**Figure 2:**
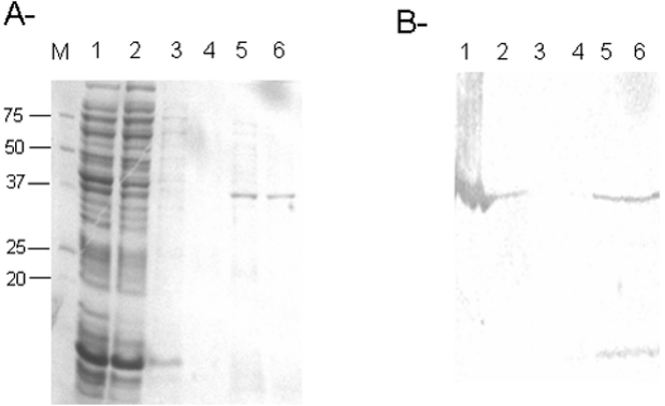
SDS-PAGE (A) and western blot (B) analysis of protein factions containing IntI1(his)_6_. Lane M: molecular weight markers in kDa; lane 1: soluble crude extract from *E. coli* DH5α expressing IntI1(his)_6_; lane 2: non-retained fraction; lanes 3, 4, 5 and 6: fractions obtained after elution with respectively 20, 100, 250 and 350 mM imidazole. Western blot was performed using anti-(his)_6_Ct antibodies (INVITROGEN).

To obtain a sufficient quantity for enzyme purification, overexpression of the IntI1 protein was performed in the BL21 *E. coli* bacterial strain at 25°C for 4 hours after 1 mM IPTG induction. At higher temperature, most of the protein remained in the insoluble fraction, reflecting the high insolubility of the protein previously observed [9], [16]. Extraction in the presence of 500 mM NaCl and 0.25% Triton X-100 allowed us to obtain a highly soluble enzyme. The soluble fraction was used for nickel-affinity chromatography purification. As shown in figure 2A, a protein displaying a good level of purity was obtained in the 250–350 mM imidazole fractions. The major protein band of 40 kDa apparent molecular weight reacted with anti-His monoclonal antibodies, thereby confirming its nature (figure 2B).

### *In vitro* binding of the recombinant IntI1 enzyme to *attI* and *attC* sites

**Figure 3:**
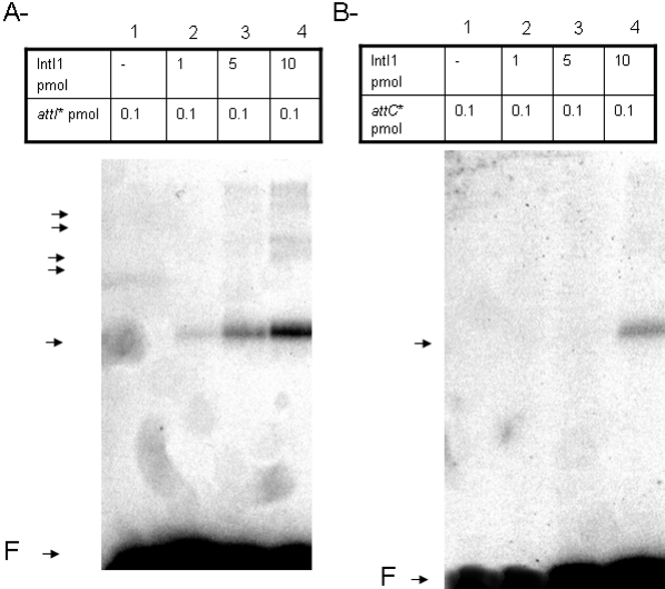
*In vitro* DNA binding of IntI1 with free double-stranded *attI1* (A) and *attC* (B) recombination sites. Free 5′ ^32^P radiolabeled dsDNA fragments containing recombination sites (0.1 pmoles) were incubated with purified IntI1 (1–10 pmoles) at 4°C for 20 min before electrophoresis on 1% agarose gel run at 50 V, for 2 hours at 4°C. Arrows indicate the protein-DNA complexes and F corresponds to free recombination sites.

To investigate the ability of IntI1 to interact with the target sites *attI1 and attC*, standard gel mobility shift assays were performed using two radiolabeled fragments containing either the double-stranded *attI1* or the *attC* site (respectively *attI1ds* and *attCds*). As shown in figure 3A, the mobility of the DNA fragment carrying the *attI1ds* site was lowered in the presence of IntI1.

The proportion of the bound substrate was dependent on the IntI1concentration. The IntI1-DNA complexes observed were consistent with those previously described using other recombinant enzymes such as MBP-IntI1 and FLAG-IntI1 [9], [10]. Even if the same IntI1-DNA complexes were detected, the intensity of the corresponding bands was different from that previously described. We assumed that this difference of affinity could be due to the different enzyme constructions used as well as differences in the DNA binding assay conditions (concentration of substrate and enzyme). In all cases the most intense band was assumed to be constituted by the target DNA and a single molecule of IntI1 and the other complexes could involve the interaction of several molecules of IntI1.

**Figure 4:**
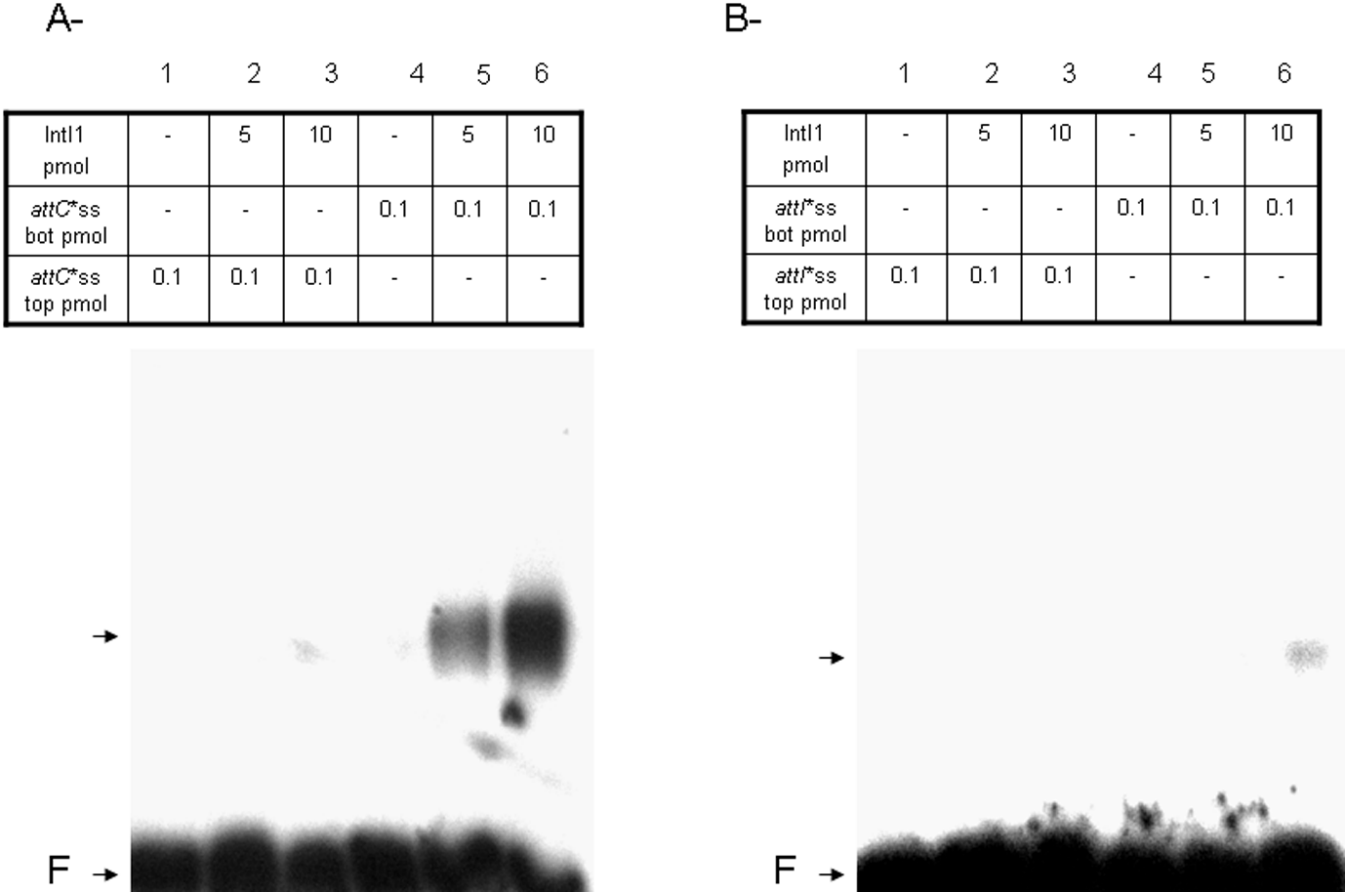
*In vitro* DNA binding of IntI1 with free single-stranded *attC* (A) and *attI* (B) recombination sites. Free 5′ ^32^P radiolabeled ssDNA fragments containing recombination sites (0.1 pmoles) were incubated with purified IntI1 (5–10 pmoles) at 4°C for 20 min before electrophoresis on 1% agarose gel run at 50 V, for 2 hours at 4°C. Arrows indicate the protein-DNA complexes and F corresponds to free recombination sites.

In contrast to this, the *attCds* fragment led to discrete complexes only at high enzyme concentrations (figure 3B). Again, the main species appeared to be the complex between one molecule of IntI1 and the DNA fragment. This suggests that the binding of IntI1 to the *attCds* element was weaker than to *attI1ds*, as previously reported [9], [10], [16]. These DNA binding data also suggest, as previously proposed, that IntI1 could bind to DNA forms different from the standard double helix. This hypothesis has been confirmed by several studies describing the specific interaction of the enzyme with ssDNA derived from the *attC* fragment [10], [15], [16]. To determine whether our recombinant IntI1 share the same property, we performed gel retardation assays using single-stranded (ss) *attI1* and *attC*. As shown in figure 4A, IntI1 specifically retarded the bottom strand of *attC* (*attCbs*) but not the corresponding top strand (*attCts*). In contrast, only a low retardation rate was observed with the single-stranded *attI1* bottom fragments (figure 4B).

**Figure 5:**
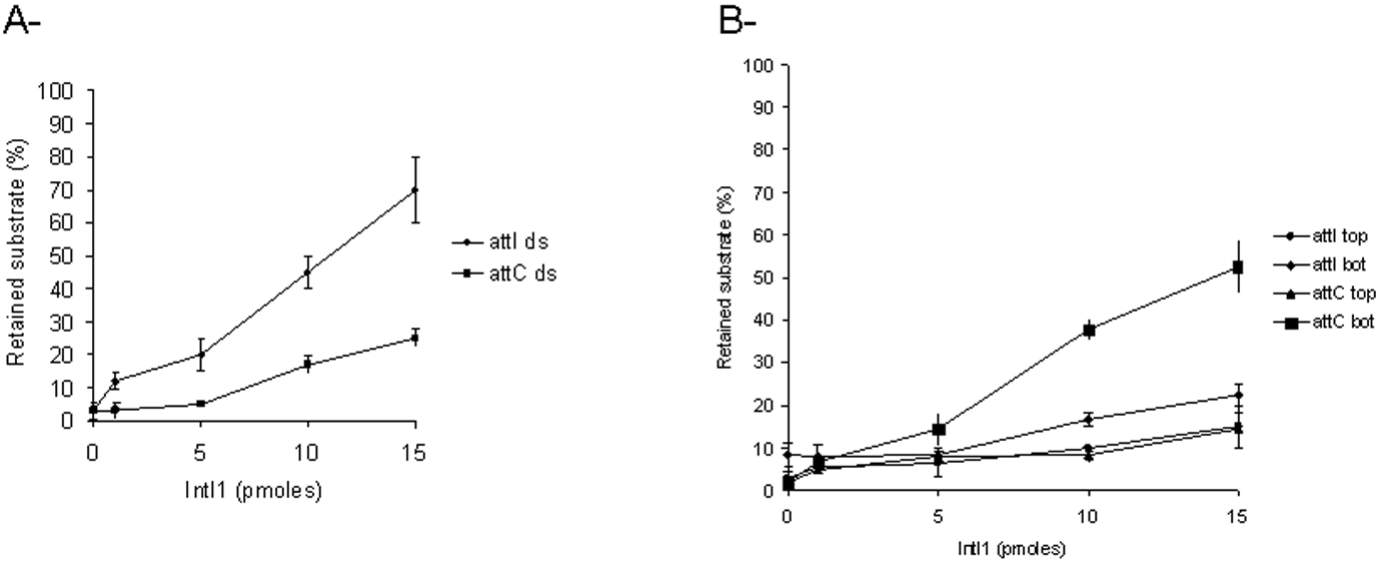
Comparison of IntI1 1 affinity for double-stranded (A) and single-stranded (B) recombination sites. Filter binding assays were performed as described in materials and methods section using either double-stranded *attI* (*attI ds*) and *attC* (*attC ds*) either top (top) or bottom (bot) strand of *attI* and *attC.* Percentages of substrate retained on filters are shown. Values are the mean±standard deviation (error bars) of three independent experiments.

Further determination of the IntI1 affinity for the recombination substrates used in this study was performed by quantitative filter binding assay. The data in figure 5 confirm the affinity of the recombinant enzyme for double-stranded *attI* and bottom single-stranded *attC* fragments. They also indicate that at high protein concentrations (above 500 nM, corresponding to 10 pmoles per assay), some unspecific binding to double-stranded *attC* and single-stranded *attI* fragments was observed (unspecific binding was also observed under these concentration conditions when an unspecific random 100 bp ODN was used, data not shown). This may indicate that at high concentration the enzyme is able to aggregate on DNA, leading to unspecific complexes.

Taken together, these results indicate that IntI1 can bind to *attI1* in a double-stranded form and to *attC* in its single-stranded form, with a bottom strand specificity as previously observed using other recombinant integrases [16]. In addition, all these data indicate that our recombinant IntI1(his)_6_ shares DNA binding properties similar to those previously described with other purified proteins. From these results we decided to study the biochemical properties of our enzyme further by setting up *in vitro* recombination assays.

### *In vitro* intermolecular strand transfer between *attI1* and *attC* sites catalyzed by the recombinant IntI1 enzyme

It has previously been reported that IntI1 can catalyze *in vivo* recombination between two *attI1*, two *attC*, or one *attI1* and one *attC*, although with varying efficiencies. In the case of class 1 integron recombinase, few data on the reaction mechanism are available due to the difficulty of obtaining a pure IntI1 that is active enough for an *in vitro* recombination assay to be performed. The lack of an easy-to-use *in vitro* system reproducing the recombination reaction further limits characterization of the catalytic mechanism and also the search for potential inhibitors. For these reasons we focused our attention on setting up an efficient *in vitro* IntI1 activity test.

The recombinant IntI1 was incubated with the radiolabeled donor substrate (either *attIds* or *attCds*) and the acceptor vector mimicking a circularized cassette (pGEM-T-*attI1* or pGEM-T-*attC*).

**Figure 6:**
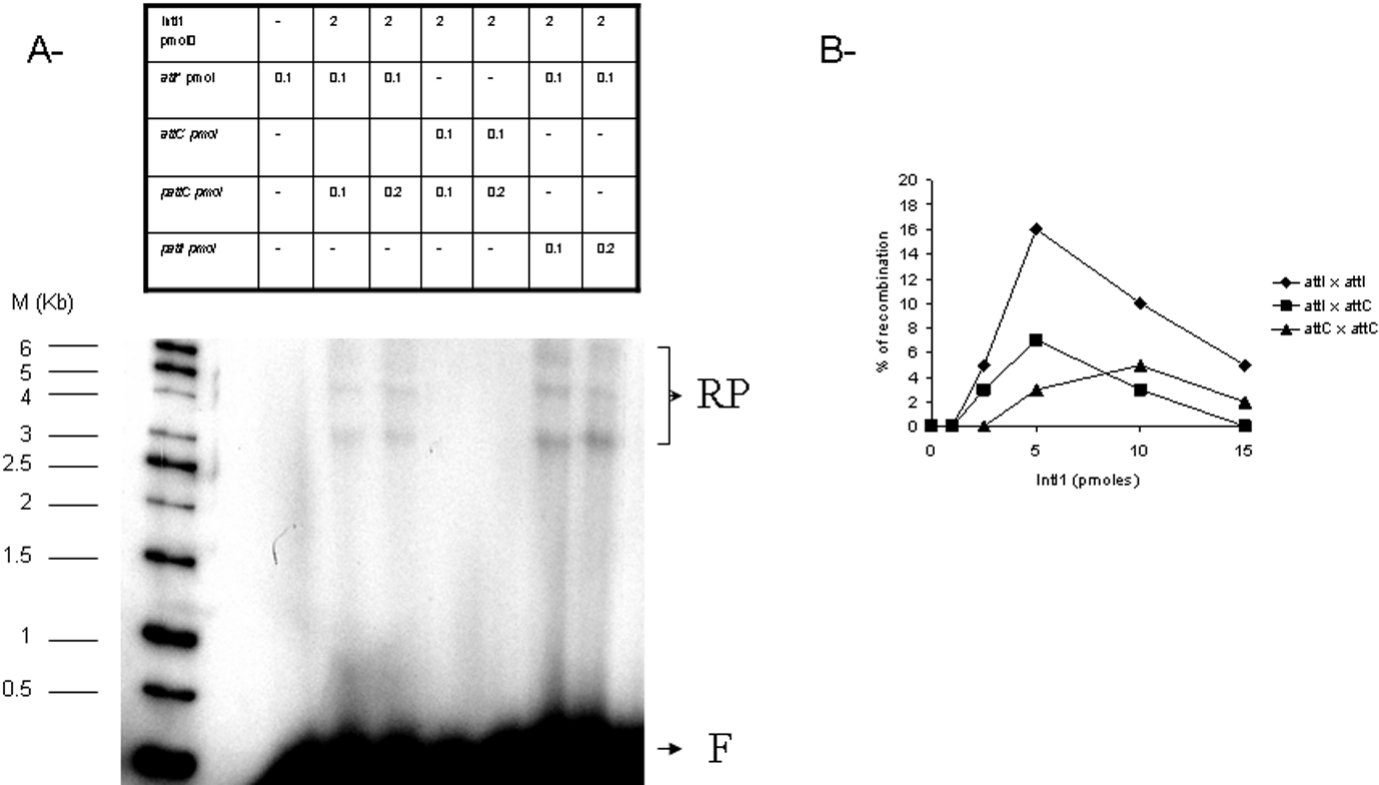
*In vitro* recombination catalyzed by IntI1 at *attI1* and *attC* sites. Reactions were performed for 90 min in the presence of purified IntI1 (5 or 10 pmoles), 0.1 to 0.2 pmoles of either pGEM-T-*attI1* or pGEM-T-*attC* (p*attI1* and p*attC* in the figure) and 0.1 pmoles free 5′ ^32^P radiolabeled recombination sites under standard conditions described in materials and methods. Products were loaded on 1% agarose gel and autoradiographied (A). F: free recombination sites, RP: recombination products. The recombination products were quantified and the percentage of recombination versus the amount of IntI in pmoles was plotted (B).

Products were detected only after proteinase K treatment of the reaction fractions, consistent with the fact that tyrosine recombinases are known to bind very tightly with DNA which is released from complexes after proteinase K digestion. After treatment a band migrating to the position expected for the recombinant product (3200 bp) was observed with both substrates, as shown in figure 6A. In addition to the expected product, other wider bands were also observed and were assumed to be intermediary recombination structures previously detected in recombination reactions catalyzed by other recombinases [20], [21].

Quantification of the reactions products showed that reaction efficiencies varied from one to another experiments, the best recombination being obtained between two *attI1* elements compared to recombination involving only one *attC* fragment (see figure 6B). This result displays some differences with the *in vivo* published data, where recombination involving two *attI1* was less efficient than the reaction involving *attC*, but is consistent with the *in vitro* DNA binding property of recombinant IntI1. Higher concentrations of protein (above 500 nM, corresponding to 10 pmoles of IntI1 per assay) led to the inhibition of recombination activity. This is also consistent with the results of the filter binding assay shown in figure 5, since under those concentrations IntI1 bound DNA unspecifically, probably leading to inactive complexes on the recombination sites. Recombination was observed between both double-stranded *attC* despite the low affinity of the enzyme for the oligonucleotide. However, in this case, a higher amount of enzyme was required (10 pmoles) compatible with the DNA binding results shown in figure 4B and 5A.

**Figure 7:**
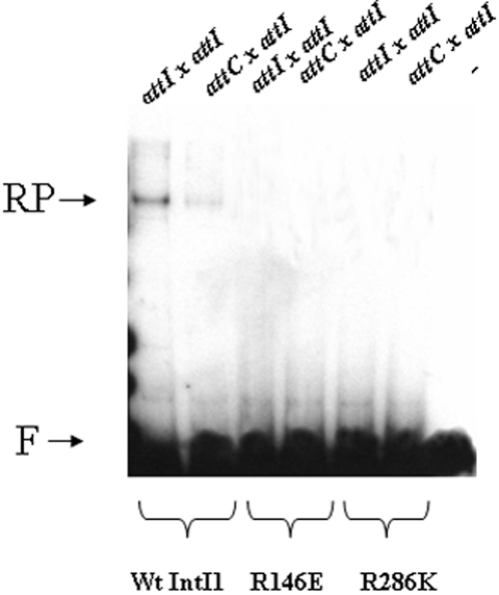
*In vitro* recombination catalyzed by wild type, R146E and R286K mutated IntI1. Reactions were performed for 90 min in the presence of purified enzyme (5 pmoles), 0.1 pmoles of linear radiolabeled recombination sites *attI1* or *attC* and 0.1 pmoles of pGEM-T-*attI1* under standard conditions described in materials and methods section. Products were loaded on 1% agarose gel and autoradiographied. F: free recombination sites, RP: recombination products.

To better ascertain the specificity of the *in vitro* recombination reaction catalyzed by IntI1, two mutated enzymes containing amino acid substitutions R146K and R280E were assayed. Those two invariant residues were demonstrated to be involved in the *in vivo* recombination activity. Since the two mutants were previously shown to be inactive for *in vivo* recombination activity [19], we analyzed their *in vitro* properties. DNA binding experiments (data not reported here) showed that only the R146K mutant presented an *in vitro* DNA binding property in the presence of *attI1* but not in the presence of the *attC* element. The second R280E mutant showed no affinity at all for *attI1* or *attC* as previously described [22]. The *in vitro* activities of both mutants were then assayed for both *attI* x *attI* and *attI* x *attC* recombinations. As shown in figure 7, no activity was detected in either mutant. These results demonstrate firstly that the two amino acids R146 and R280 are required for recombination catalysis, and that the reaction observed with wild type IntI1 was due to the intrinsic catalytic capability of the recombinant integrase.

### *In vitro att*C recombination involves the bottom single strand of the recombination fragment

**Figure 8:**
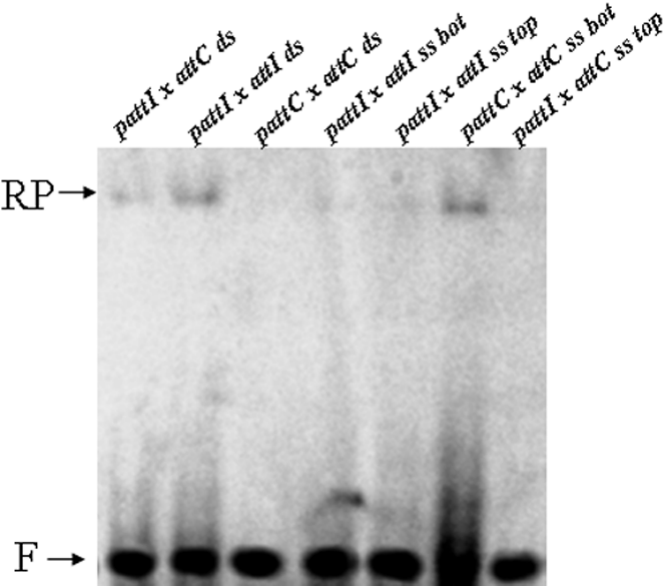
*In vitro* recombination catalyzed by wild type IntI1 in presence of double- or single-stranded substrates. Reactions were performed for 90 min in the presence of purified enzyme (5 pmoles), 0.1 pmoles of linear radiolabeled double-stranded (ds) or single-stranded (bottom strand: ss bot, or top strand: ss top) recombination sites *attI1* or *attC* and 0.1 pmoles of pGEM-T-*attI1* or pGEM-T-*attC* under standard conditions described in materials and methods section. Products were loaded on 1% agarose gel and autoradiographied. F: free recombination sites, RP: recombination products.

As reported above (figure 6) recombination activity was detected even in the presence of double-stranded *attC*. Since it has been proposed that IntI1 recombination could involve the bottom single strand of the *attC* site, we further analyzed the *in vitro* activity of IntI1 using single-stranded ODNs. Figure 8 shows that the only recombination product detected with the single-stranded substrate was in the presence of the bottom strand of *attC.* Therefore, the reaction involving the single-stranded bottom strand of *attC* was the most effective under our conditions. No recombination products were detected in the presence of single-stranded *attI*, strongly suggesting that these events do not share the same mechanism as *attC* recombination. These results are in agreement with previous reports [14] and confirm that *attC* recombination requires the bottom *attC* strand.

### Biochemical parameters of the *in vitro* recombination reaction

**Figure 9:**
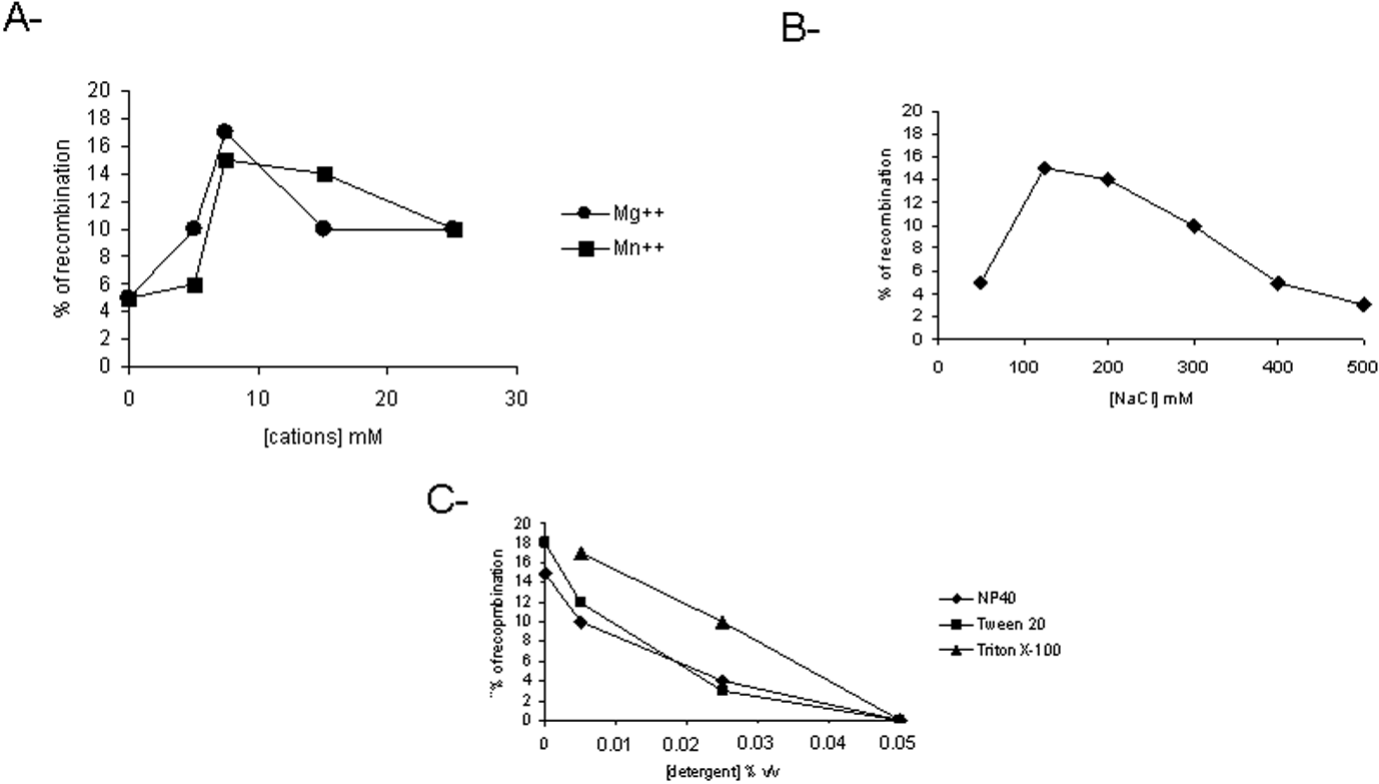
Effect of cations (A), salt (B) and detergent (C) on *in vitro* recombination catalyzed by IntI1. Recombination reactions were performed in the presence of 2 pmoles purified IntI1, 0.1 pmoles free attI1^*^ and 0.1 pmoles pGEM-T-*attI1* and different concentrations of MgCl_2_, MnCl_2_ NaCl and detergent as indicated. Recombination products were quantified with DNAJ software and are shown on the graphs as the percentage of recombinant product versus the total substrate.

To optimize the reaction conditions, we tested the requirement of the enzyme for cations (Mg^++^ and Mn^++^) and NaCl. As shown in figure 9A, IntI1 can use both cations for recombination with an optimum at 7.5 mM Mg^++^. Importantly, a basal recombination activity was detected under our conditions even in the absence of cations in the reaction medium. The NaCl concentration was found to affect dramatically the recombination activity of IntI1 (figure 9B). A 125 mM concentration was required for optimal activity and the recombination reaction was inhibited at higher salt concentrations. We also investigated whether the addition of small amounts of detergent could improve the activity. Three molecules were tested: Np40, Tween 20 and Triton X-100. Figure 9C shows that all of them inhibited the recombination reaction at different levels, as recently reported for the yeast FLP recombinase belonging to the same tyrosine recombinase family [23].

## Discussion

Gene cassette mobility in integrons in bacteria is a principal factor in the spread of antibiotic resistance, thereby reducing the efficiency of long-term antibiotherapy. IntI1 has previously been put forward as the enzyme responsible for this mobility. The availability of a pure *in vitro* active integrase is the prerequisite for detailed examination of the recombination reactions catalyzed by this enzyme. The purpose of our work was to set up an *in vitro* assay allowing the detection of junction molecules from the recombination activity catalyzed by IntI1 on *att* fragments and thus the further characterization of its biochemical properties.

IntI1 was expressed and purified as a protein fused to (his)_6 _tag in C-terminal. The methodology presented here, in particular the use of a high salt concentration, allowed us to overcome the main obstacle to integrase purification, i.e. its very high insolubility [9], [16].

*In vitro* DNA binding analysis using free double-stranded *attI1* and *attC* fragments indicated that IntI1 shared the same property as previously described purified enzymes such as native integrase, MBP-IntI1, Flag-IntI1 [9], [10]. In particular, IntI1 bound *in vitro* to double-stranded *attI1* with a higher efficiency than to double-stranded *attC* as previously reported [9], [10], [16]. In contrast, IntI1 showed a better affinity for the bottom strand of *attC* than its opposite top strand or single strands derived from *attI*. These results confirmed the previously reported data indicating that the enzyme recognizes a preferred ssDNA structure in the *attC* recombination site. Some unspecific binding was also observed at high protein concentration, suggesting the formation of aggregates on DNA. To better determine the relationship between DNA affinity of IntI1 and its recombination activity, we used the *in vitro* assays developed in this study.

Intermolecular recombination reactions using free *attI1* and *attC* target sites showed purified IntI1 could catalyze at least a strand transfer *in vitro*. This result demonstrates that IntI1 is necessary and sufficient for basal recombination between *attI* or *attC* sites in class 1 integron. Indeed, IntI1 has been previously put forward as the enzyme responsible for the movement of gene cassettes in and out of integrons, but no data suggested conclusively that it was the sole protein involved in cellular recombination events. Other proteins belonging to the same family need cellular factors to perform their activity efficiently. For example, excision of lambda prophage from the bacterial chromosome requires the host-encoded integration factor (IHF) and the lambda-encoded excisionase in addition to integrase and both integration and excision are stimulated by the host-encoded factor for inversion stimulation (FIS) protein [24]. Our data provide the first evidence that IntI1 possesses all the catalytic activities needed to perform the first strand transfer step between recombination sites.

However, differences were observed in recombination efficiency depending on the target DNA sites. In general, *in vitro* recombination between two *attI1* was preferred over recombination between *attI1* and *attC* sites, itself more efficient than between two *attC*. This result is in agreement with the *in vitro* DNA binding property of the recombinant IntI1 and can be explained by a lower *in vitro* affinity of IntI1 with the *attC* element compared to *attI*. This suggests a preference for the specific structure found in *attI1*. Our results differ somewhat from those reported in the literature. Indeed, *in vivo* recombination events between two *attI1* and *attC* sites are more efficient than those involving two *attC, which* themselves are more efficient than *attI* x *attI* recombination [13]. Differences observed between *in vivo* and *in vitro* suggest that the integration mechanism in the cell might be regulated, favoring the recombination between *attI1* and *attC*. Under our conditions we did not observe *in vitro* integration at secondary sites when using the receptor plasmid lacking *att* sites in the recombination assay (data not shown), thus confirming the low frequency of those events.

Biochemical analysis of the recombination reaction catalyzed by IntI1 showed that the enzyme could perform its activity in the absence of bivalent cations added to the reaction mixture at a basal level but preferring a 7.5 mM Mg^++^ concentration (see figure 7). Although there is disagreement regarding the effect of ions on recombination, there is a consensus that divalent ions are required for the intermolecular DNA exchange reaction [25]–[27]. Our observation raises the question whether cations are coordinated in the integrase structure and play a role in the reaction. In addition, we showed that IntI1 activity was highly sensitive to detergents and, at a lower level, to salt. All the tested detergents (Np40, Tween 20 and Triton X-100) inhibited the recombination reaction, as recently reported for other tyrosine recombinase [23]. This indicates the possible involvement of oligomeric forms of the enzyme in the reaction, as previously demonstrated for enzymes belonging to the same family [28]. Such active oligomers could be dissociated by detergent and high salt concentration inhibiting the recombination reaction. This assumption is reinforced by the DNA protein complexes observed between IntI1 and the free purified recombination sites by us and others [9], [16]. Nevertheless, we cannot rule out a possible dissociation between IntI1 and recombination sites since salt can also weaken such interactions.

The *in vitro* recombination data indicate that *attI1* and *attC* sites do not interact equally with IntI1, thereby confirming that there are different mechanisms for recombination depending on the sites involved. The difference in affinity of the enzyme for *attI1* and *attC* and the preference of IntI1 for single-stranded *attC* previously reported [16] and also observed by us strongly support the involvement of a single-strand DNA intermediary in the reaction, as recently proposed [14]. To better ascertain this requirement, we performed assays with single-stranded substrates. Our results indicate that *in vitro attC* recombination requires the bottom strand of the site in contrast to *attI* recombination. Our data unambiguously demonstrate that *attC* and *attI* recombination mechanisms do not share the same process.

Nevertheless, in all cases only a maximum of about 15–18% of the donor substrate was integrated into the acceptor DNA, indicating that the *in vitro* recombination still had a low level of efficiency. Whether this efficiency reflects that of *in vivo* recombination remains to be established. This low level of recombinative integration could be due to the fact that two independent molecules are involved in this *in vitro* reaction. Furthermore, the DNA fragments used in our assay do not share the exact structure of the total integron where intermolecular recombination takes place. In addition, the differential affinity of IntI1 for *attI1* and *attC* free fragments previously observed could explain the variation in recombination efficiency.

How can our *in vitro* recombination data be reconciled with the specificity of IntI1 for single-stranded *attC*? The recombination activity observed in presence of the bottom strand of *attC* confirms that this single-stranded structure is an important intermediary in the reaction, as previously reported [14], [15]. Moreover, an *in vitro* recombination activity between double-stranded *attI* and *attC* and between two double-stranded *attC* could also be detected, implying that the enzyme was able to generate and/or recognize the single-stranded structure in the *attC* site, even in the absence of other bacterial factors usually involved in this kind of mechanism such as helicase. Recently it has been shown that integron integrase binds to bulged hairpin DNA found at the *attC* site [17]. These cruciform structures could be generated *in vivo* by a cellular mechanism such as DNA replication and transcription and then stabilized by IntI1 for recombination. The capacity of the enzyme to generate the single strand by itself should allow the recombination to be effected independently of the replication processes. However, in standard helicase assays performed with our pure fractions of IntI1, no activity was shown (data not shown). This suggests that generation of the single-stranded *attC* by the enzyme acts via a different way, as suggested by the structural data [15], probably by strong interaction between IntI1 and the extrahelical bases in the recombination site, leading to opening of the strands.

By dissecting the reaction, the recombination assays described will allow further characterization of this activity. In particular, the recombination test could serve as a basis for the selection and the study of inhibitory molecules as well as for the analysis of reaction selectivity using chimeric proteins. These could be useful tools in gene therapy. The results obtained with the mutated enzyme also indicate that the *in vitro* assays described here make it possible to study the relationship between the structure and function of IntI1 through analysis of the biochemical properties of the mutation carrying integrases in amino acids, which is potentially involved in recombination. In addition to such fundamental studies the *in vitro* assay described here will allow the search and the selection of specific inhibitors of IntI1 that could be useful for the *in vivo* limitation of the antibiotic resistance spread.

## Materials and Methods

### DNA, bacterial strains and culture media

The DNA sequence encoding the entire class I integron was previously cloned from *P. aeruginosa* Pa695 [18] into pC23 vector (see figure 1). The natural conjugative plasmid pSf2032 which contains a class 2 integron carrying trimethoprime (cassette 1), streptothricin (cassette 2) and streptomycin-spectinomycin (cassette 3, silent gene) resistance genes was obtained from the *Shigella flexneri* Sf2032 clinical strain. The pACYC184-*attI1* vector, carrying a chloramphenicol resistance determinant and an *attI1* site, was obtained by ligating the PCR amplification product, on one hand, obtained with pC23 as template and attI1L-BamH1 (5′-GGATCCCAAGCAGCAAGCGCGTTAT-3′) and attI1R-HindIII (5′-AAGCTTGCAATGATGCTCATTGAGCC-3′) as primers, cut with *Bam*HI and *Hind*III and, on the other, pACYC184 with the same enzymes.

The *E. coli* DH5α strain was used for propagation of plasmids and *E. coli* BL21 strain was used for expression of IntI1(his)_6_ recombinant enzyme. *E. coli* TOP10 was the recipient strain in *in vivo* recombination assays. All bacterial strains were cultured at 37°C for propagation of the plasmid or 25°C for expression, on LB medium supplemented with antibiotic.

### DNA manipulation

All DNA vectors and PCR products were purified using the DNA purification systems from PROMEGA (Wizard plus SV miniprep and Wizard SV Gel kits). PCR amplifications were done under standard conditions using *Taq* polymerase (PROMEGA). Sequencing was performed by polymerase chain reaction-based sequencing (ABI Prism big dye terminator cycle sequencing ready reaction kit, Applied Biosystems).

#### Cloning, expression and purification of IntI1

Wild type *IntI1* gene was amplified by PCR from pC23 vector using IntI1-5′-Topo primer containing the Topo1 site (5′-CACCATGAAAACCGCCACTGCGCCG-3′) and IntI1-3′-stop primer (5′-CCTCTCACTAGTGAGGGGCGGCAG-3′). The amplification product was then cloned into pET101D-Topo vector according to the manufacturer's recommendations (INVITROGEN). The resulting pET101D-IntI1 expression plasmid was checked by sequencing and introduced into the BL21 bacterial strain for expression of recombinant IntI1 fused to (his)6 tag at the 3′-end. The genes encoding the mutated R146K and R280E enzyme were amplified as for wild type enzyme but from pMalC2 plasmid kindly provided by Drs. P. Roy and N. Messier [20]. Both mutants were further cloned into pET101D vector as done for wild type enzyme.

Bacteria containing the pET101-IntI1 vector were cultured in LB medium supplemented with ampicillin (50 µg/ml) for 12 hours at 37°C with shaking. Cultures were then diluted at 1/60 in fresh LB-ampicillin (50 µg/ml) and cultured for 4 more hours to reach an OD_600 nm_ of 0.4. Expression of IntI1 was induced by addition of IPTG to 1 mM for 4 hours at 25°C, since at 37°C a major proportion of the enzyme remains insoluble. Cells were harvested by centrifugation at 4000 rpm for 15 minutes and pellets were resuspended in 5 ml of lysis buffer I (50 mM NaH_2_PO_4_ pH 7.5) containing a cocktail of proteases inhibitors (Complete Mini EDTA free, ROCHE) and 50 µg/ml of lysozyme. After 20 min incubation at room temperature, the suspension was centrifuged for 15 minutes at 4000 rpm. Pellets were resuspended in 5 ml of lysis buffer II (50 mM NaH_2_PO_4_ pH 7.5, 500 mM NaCl, 1 mM DTT, 0.025% Triton X-100). The suspension was incubated for 15 min at 4°C, and then sonicated. The cell lysate was centrifuged at 18000 rpm for 20 min at 4°C and the supernatant was used for SDS-PAGE analysis and purification. With the procedure described above, the majority of IntI1 could be recovered in a soluble and thus suitable form for purification.

A 600 µl aliquot of the soluble fraction obtained as described above was loaded on a Ni-NTA Spin Column according to the supplier's instructions (QIAGEN). The column was washed twice with the elution buffer (50 mM NaH_2_PO_4_ pH 7.5, 500 mM NaCl, 0.025 Triton X-100) containing 20 mM imidazole. Several elution steps were then performed from 20 mM to 400 mM imidazole. The fractions were analyzed by SDS-PAGE after staining with blue Coomassie and western blot using anti-(His)_6_Ct antibodies (INVITROGEN). The fractions containing IntI1 were dialyzed against 50 mM Na_2_HPO_4_/NaH_2_PO_4_ pH 7.5, 500 mM NaCl, 0.025 Triton X-100 solution and then stored at −20°C after addition of 10% glycerol. Protein concentrations were determined by the standard Bradford method. Stock concentrations varied from 2.5 to 5 µM.

#### *In vivo* recombination assay

An *in vivo* excision assay was performed using DH5α cells containing the pSF2032 and pET101D-IntI1 plasmids. The strain was cultured in 5 ml LB medium supplemented with ampicillin (100 µg/ml ) and trimethroprime (20 µg/ml) for 4 hours at 37°C under shaking. Then, 1 mM IPTG was added, and after 3 and 24 hours of culture, 10 µl aliquots were plated on LB solid medium containing ampicillin (100 µg/ml) and trimethoprime (20 mg/ml). After 18 hours of incubation at 37°C, 10 clones were recovered and analyzed by PCR using primers int2inv (5′-AACCTTTTTGTCGCATATCCGTG-3′), dfrA1R (5′-GTTAGAGGCGAAGTCTTGGG-3′), satinv (5′-TTAGGCGTCATCCTGTGCTC-3′), and aad2di (5′-CAGGAACCGGATCAAAGAG-3′) to determine the number of gene cassettes in the integron. A DH5α strain carrying only pSf2032 served as control.

An *in vivo* recombination assay was carried out using DH5α cells harboring the pSf2032, pACYC184-*attI1* and pET101D-IntI1 vectors as the donor strain, and TOP10 as the recipient strain in a filter matting assay. Both strains were cultured in LB medium supplemented or not with antibiotics (100 µg/ml ampicillin, 20 µg/ml trimethoprime and 30 µg/ml chloramphenicol) for 3 hours at 37°C under shaking, and then for 3 more hours after addition of 1 mM IPTG to induce IntI1 expression. Cultures of each strain were concentrated by centrifugation, and 1 ml aliquots were loaded on Millipore GS 0.22 µm filter, then plated on LB medium. After 16 hours incubation at 37°C, transconjugants were selected on LB medium supplemented with trimethoprime (20 µg/ml), streptomycin (25 µg/ml) and chloramphenicol (30 µg/ml), or with trimethoprime and streptomycin only. Donor cells were numbered to determine the transfer frequency and recombination rate was calculated as the ratio between the number of transconjugants containing the recombinant plasmid versus the total number of transconjugants.

#### *In vitro* strand transfer assays

The *attI1* containing donor substrate (100 bp) was generated by annealing the 5′^32^P radiolabeled oligonucleotide AttI (5′-ACGCCGTGGGTCGATGTTTGATGTTATGGAGCAGCAACGATGTTACGCAGCAGGGCAGTCGCCCTAAAACAAAGTTAGGTGGCTCAATGAGCATCATTGC-3′) with the complementary one AttI'. The *attC* containing donor substrate (120 bp) was obtained by annealing the 5′^32^P radiolabeled oligonucleotide AttC2 (5′-CGCCCGTCTAACAATTCGTTCAAGCCGACGTTGCTTCGTGGCGGCGCTTGCGTGCTACGCTAAGCTTCGCACGCCGCTTGCCACTGCGCACCGCGGCTTAACTCAGGCGTTAGATGCACT-3′) with the complementary one AttC2′.

To construct the receptor plasmids pGEM-T-*attI1* (called p*attI1*) and pGEM-T-*attC* (called p*attC*), both *attI* and *attC2* were generated by PCR using attI1-LBamH1 and attI1-RHindIII as primers and pC23 as template (see figure 1). PCR led to the amplification of the *attC* site of the second cassette [*attC(2)* in figure 1]. The receptor plasmids pGEM-T-*attI1* (called p*attI1*) and pGEM-T-*attC* (called p*attC*) were obtained by cloning the corresponding fragments into pGEM-T easy vector (PROMEGA).

To determine its DNA binding activity, purified IntI1(his)_6_ (1 to 10 pmoles) was incubated either with the 5′ radiolabeled double-stranded or single-stranded *attI1* fragment or with the 5′ radiolabeled double-stranded or single-stranded *attC* fragment for 20 min at 4°C in a total volume of 20 µl. The IntI1-DNA complexes were then loaded on vertical 1% agarose gel and run at 50 V for 4 hours at 4°C. The gel was then dried and autoradiographied. Quantification was performed by filter binding assays: Nitrocellulose filters (0.45 µm, Whatman) were treated with a solution of KOH 0.4 M and washed twice with water and 2 ml of pre-washing buffer (HEPES 20 mM; pH 7.5; MnCl_2 _10 mM; NaCl 10 mM; calf thymus DNA 100 µg/ml). IntI1 was incubated under *in vitro* assay conditions for 20 min at 4°C with the different radiolabeled substrates. After addition of 1 ml washing buffer (HEPES 20 mM; pH 7.5; MnCl_2_ 10 mM; NaCl 30 mM) to the reaction mix, the solution was filtered. Filters were washed twice with 4 ml of washing buffer. The radioactivity retained on filters was quantitated with a scintillation counter (Wallac 1409).

The recombination reaction was performed by incubation of the purified IntI1(his)_6_ (1 to 10 pmoles) with both donor and receptor substrates (0.1 to 0.2 pmoles) for 20 min at 4°C in a total volume of 5 µl to promote IntI1-DNA complexes. Then, the incubation proceeded at 37°C for 90 min in the presence of 7.5 mM Mg^++^, 50 mM TrisHCl pH 7.5 and 1 mM DTT in a total volume of 20 µl. Reaction fractions were treated by protease K (50 µg/ml) for one hour at 55°C and were then submitted to phenol/chloroform/isoamylalcohol (25/25/1, v/v/v) extraction. The aqueous fraction was loaded on vertical 1% agarose gel and run at 200 V. The gel was dried and autoradiographied.
